# Using Y-Chromosomal Haplogroups in Genetic Association Studies and Suggested Implications

**DOI:** 10.3390/genes9010045

**Published:** 2018-01-22

**Authors:** A. Mesut Erzurumluoglu, Denis Baird, Tom G. Richardson, Nicholas J. Timpson, Santiago Rodriguez

**Affiliations:** 1 Genetic Epidemiology Group, Department of Health Sciences, University of Leicester, Leicester LE1 7RH, UK; ame26@leicester.ac.uk; 2 MRC Integrative Epidemiology Unit (IEU), Population Health Sciences, Bristol Medical School, University of Bristol, Oakfield House, Oakfield Grove, Bristol BS8 2BN, UK; denis.baird@bristol.ac.uk (D.B.); tom.g.richardson@bristol.ac.uk (T.G.R.); n.j.timpson@bristol.ac.uk (N.J.T.)

**Keywords:** Y-DNA, haplogroups, body mass index, Avon Longitudinal Study of Parents and Children, 1958 Birth Cohort

## Abstract

Y-chromosomal (Y-DNA) haplogroups are more widely used in population genetics than in genetic epidemiology, although associations between Y-DNA haplogroups and several traits, including cardiometabolic traits, have been reported. In apparently homogeneous populations defined by principal component analyses, there is still Y-DNA haplogroup variation which will result from population history. Therefore, hidden stratification and/or differential phenotypic effects by Y-DNA haplogroups could exist. To test this, we hypothesised that stratifying individuals according to their Y-DNA haplogroups before testing for associations between autosomal single nucleotide polymorphisms (SNPs) and phenotypes will yield difference in association. For proof of concept, we derived Y-DNA haplogroups from 6537 males from two epidemiological cohorts, Avon Longitudinal Study of Parents and Children (ALSPAC) (*n* = 5080; 816 Y-DNA SNPs) and the 1958 Birth Cohort (*n* = 1457; 1849 Y-DNA SNPs), and studied the robust associations between 32 SNPs and body mass index (BMI), including SNPs in or near Fat Mass and Obesity-associated protein (*FTO*) which yield the strongest effects. Overall, no association was replicated in both cohorts when Y-DNA haplogroups were considered and this suggests that, for BMI at least, there is little evidence of differences in phenotype or SNP association by Y-DNA structure. Further studies using other traits, phenome-wide association studies (PheWAS), other haplogroups and/or autosomal SNPs are required to test the generalisability and utility of this approach.

## 1. Introduction

The interpretation of genetic association studies (including candidate gene studies and genome-wide association studies, (GWAS)) requires consideration of many potential confounders including population stratification, gene-gene interaction and gene-environment interaction [1,2,3]. The relevance of these factors, in particular population structure and haplotype background [4], has been explored by the analysis of autosomal markers. In contrast, non-recombining genetic variation such as Y-chromosomal (Y-DNA) haplogroups, has rarely been considered in the design and interpretation of genetic association studies—although there are examples including direct testing of the association between Y-DNA haplogroups and phenotypes, including cardiometabolic diseases [5,6,7,8,9,10,11,12].

Analyses of the non-recombining regions of the Y chromosome in different populations provide genealogical and historical information [13,14]. Y-chromosomal lineages, through the analysis of short tandem repeats (STR), have proven useful when determining whether two apparently unrelated individuals descend from a common ancestor in recent history (<20 generations). However, using of modern genotyping arrays coupled with extensive and publicly available Single Nucleotide Polymorphism (SNP) data, researchers can now suggest which ancient ethnic group to one’s paternal ancestor belonged to. Comprehensive SNP data also enabled the construction of well-established Y-DNA haplotypes and genealogical trees [15,16,17]. This is why genetic variation in this uniparentally inherited chromosome can be used to define groups of Y-DNA haplotypes which share a common ancestor with a SNP mutation.

Haplogroups derived from Y-chromosomal variation can be used to provide information about the paternal ancestry of an individual and population genetic events (e.g., migrations, bottle necks) [18,19,20]. Genealogical relationships between haplogroups are well-known and there is wide-spread knowledge of the frequency and the type of haplogroups present in almost all geographical regions throughout the world. For example, the Y-DNA haplogroup R1b (R-M343) is frequent in Europe and infrequent or absent in other continents/sub-continents.

Facilitating the process of linking haplotype assignment to GWAS studies, there is comprehensive information about the SNPs which define each haplogroup, approximate time and (most probable) region of origin, (current) area of highest frequency and the most prevalent (ancient) haplogroup present in different regions.

Even for apparently homogeneous populations according to principal component analyses (PCA) utilising autosomal SNPs, there is underlying Y-DNA haplogroup variation. We have previously analysed Y-DNA haplotypes in a large epidemiological cohort in relation to confounding by genetic subdivision [21].

In the present work, we stratify groups of individuals according to their Y-DNA haplogroups to (i) test for presence of additional structure due to Y-DNA haplogroup variation having taken into account PCA using autosomal markers; and if there is (ii) test if this additional structure has any potential confounding effects on genetic association studies (e.g., direct association, epigenetic, epistasis). As a proof of concept, we chose to study the association between 32 common SNPs which are known to be robustly associated with body mass index (BMI). The use of these common genetic variants limits our analyses to the largest BMI effect loci.

## 2. Materials and Methods

### 2.1. Participants and Ethics

The Avon Longitudinal Study of Parents and Children (ALSPAC) is a longitudinal, population-based birth cohort study that initially recruited >13,000 pregnant women residing in Avon, United Kingdom, with expected dates of delivery between 1 April 1991 and 31 December 1992. There were 14,062 liveborn children. The study protocol has been described previously [22,23] and further details are available on the ALSPAC website [24]. Please note that the study website contains details of all the data that is available through a fully searchable data dictionary [25].

Height and weight measurements were performed on children who attended a 9-year focus group clinic (mean age of participant = 9 (±0.32 years)). Ethical approval for all aspects of data collection was obtained from the ALSPAC Law and Ethics Committee (institutional review board 00003312, Initial Approval 28th November 1989). Written informed consent for the study was obtained for genetic analysis.

The National Child Development Study (NCDS), otherwise known as the 1958 British birth cohort (1958BC), started as a perinatal mortality and morbidity survey looking at all births in England, Wales and Scotland in a single week in 1958. This included an original sample of 17,638 births (in addition to a further 920 immigrants born in the same reference week). Cohort members were further followed-up by medical examinations (at 7, 11 and 16 years of age) and interviews (at ages 23, 33 and 42). The first biomedical assessment was conducted between September 2002 and March 2004 by trained nurses from the National Centre for Social Research, who visited the homes of cohort members at age 44–45 years [26].

### 2.2. Genotyping and Imputation

#### 2.2.1. ALSPAC

A total of 9912 children (male and female) were genotyped using the Illumina HumanHap550 quad (Illumina, Essex, UK) genotyping array by 23 and Me. The Wellcome Trust Sanger Institute (Cambridge, UK), and the Laboratory Corporation of America (Burlington, NC, USA) were subcontracted. Information on the genotyping array can be found on: HumanHap550-Quad+ BeadChip [27]. PLINK software (v1.07) was used to carry out quality control (QC) measures [28]. Individuals were excluded from further analysis on the basis of having incorrect gender assignments, minimal or excessive heterozygosity (<0.320 and >0.345 for the Sanger Institute data and <0.310 and >0.330 for the Laboratory Corporation data), disproportionate levels of individual missingness (>3%), evidence of cryptic relatedness (>10% identical by descent, IBD) and being of non-European ancestry (as detected by a multidimensional scaling analysis seeded with Haplotype Map (HapMap) 2 individuals) [29]. EIGENSTRAT analysis revealed no additional obvious population stratification in relation to CEU individuals (Utah residents with Northern and Western European ancestry from the Centre d’Etude du Polymorphisme Humain collection) from phase 2 of the HapMap project (hg18) and genome-wide analyses with other phenotypes indicated a low λ [29,30]. Imputation was carried out using the Markov Chain Haplotyping (MaCH) software [31] with CEU individuals from phase 2 of the HapMap project (hg18) as a reference set (release 22).

#### 2.2.2. 1958 Birth Cohort

A total of three thousand individuals were genotyped on the Illumina 1.2M chip (Illumina) [Dataset ID: EGAD00000000022]. The Illumina 1.2M genotyping array is a customised version of the Illumina Human1M-Duo BeadChip for which information can be found on: Human1M-Duo BeadChip [32]. Quality control measures were as described above. No imputation was carried out on this dataset as rs8050136 was the only SNP analysed in the 1958BC dataset and dosage data was available.

### 2.3. Y-DNA Haplogroup Determination

For Y-DNA haplogroup determination in ALSPAC, the Y-chromosomal SNPs of all 5085 male participants in the dataset were used. The pseudo-autosomal SNPs were removed using the PLINK software package [28]. The resulting Y-chromosomal genotype (816 SNPs) of each individual was then piped in to the YFitter (v0.2) software which maps genotype data to the Y-DNA genealogical tree built by Karafet et al. [16] (Yfitter, https://sourceforge.net/projects/yfitter/), and their respective Y-DNA haplogroup was determined. After removal of individuals with ‘False’ haplogroup determinations (i.e., ones which did not have enough SNPs to reliably determine haplogroup), we were left with 5080 individuals. Remaining individuals with a haplogroup result which began with the letter R (e.g., R1b1) were clustered in to a single group named ‘Clade R’, and likewise the same was done with the haplogroups beginning with the other letters. The same procedure was carried out in 1958BC and 1453 male participants’ haplogroups were determined. Only the clades (major haplogroups) R and I were considered in the analyses, since there was not enough power for the less frequent haplogroups.

### 2.4. Association Study between Y-DNA Haplogroups and BMI

To check for association between BMI and the Y-DNA haplogroups in ALSPAC, a linear regression analysis was carried out using haplogroup R as a baseline (coded 0) and coding haplogroup I as 1. Age, age^2^ and the top 10 principal components (PCs) determined by the EIGENSTRAT software [29] were used as covariates in the model.

The analysis was repeated in the 1958BC during the replication stage. Production of summary statistics for the two cohorts and all regression analyses were carried out in the STATA statistical package [33].

### 2.5. Analysis of the Effects of Y-DNA Haplogroup on SNPs Associated with BMI in ALSPAC

In order to study whether well-established associations are still present and/or observable within each Y-DNA haplogroup and whether the effect sizes of the SNPs were consistent across the different Y-DNA haplogroups, we analysed 32 common autosomal SNPs previously reported to be associated with BMI [34]. This enables the analysis of common genetic variation with the largest effects for BMI. All individuals with missing and/or incorrectly measured data were excluded. Individuals with ‘False’ haplogroups (as determined by YFitter) were also removed. At the end of the QC procedure, 2800 individuals had complete haplogroup, BMI and genotype data in ALSPAC. Finally, individuals belonging to haplogroups with frequencies less than 1% were also excluded.

Body mass index data did not follow a normal distribution and inverse rank transformation was used to transform the data. Single Nucleotide Polymorphism dosage values were determined using MaCH [31]. A linear regression analysis between BMI and each of the 32 SNPs was carried out using STATA controlling for age, age^2^ and the first 10 PCs determined by the EIGENSTRAT software [29]. We looked at the PCA adjusted results only in order to analyse the Y-DNA haplogroup sub-structure. We checked for normal distribution of BMI within the two most frequent Y-DNA haplogroup clades observed (i.e., R and I); and also confirmed that the allele frequencies of the autosomal SNPs analysed were similar across the haplogroups. A subgroup analysis was carried out within the Y-DNA haplogroups R and I. Any possible interaction between genotype and Y-DNA haplogroup in the analyses were assessed using a likelihood ratio test to compare the two regression models, one which was adjusted for the covariates abovementioned and the Y-DNA haplogroup, and another which additionally included an interaction term (i.e., genotype x Y-DNA haplogroup). Although low powered compared to the likelihood ratio test, a heterogeneity test (*z*-test) was carried out across the two haplogroups to check whether there was a difference in the effect sizes (β coefficient) of the 32 SNPs tested.

In agreement with the Y-DNA genealogical tree, we also analysed I and J individuals together as a sensitivity analysis for the rs8050136 (in Fat Mass and Obesity-associated protein (*FTO*)) SNP.

The above methods were also used during the replication stage which utilised the 1958BC dataset.

## 3. Results

**Figure 1:**
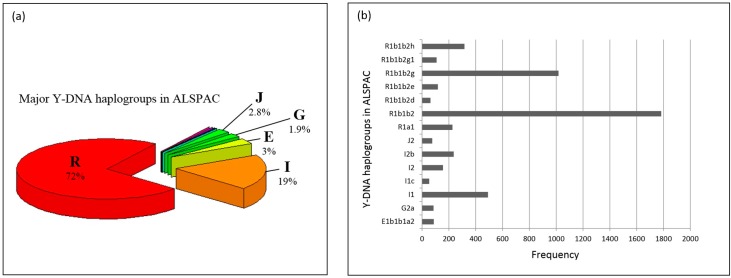
(**a**) Y-DNA haplogroups in Avon Longitudinal Study of Parents and Children (ALSPAC). ALSPAC had individuals belonging to 12 of the major Y-DNA haplogroups (C, E, G, H, I, J, L, N, O, Q, R, T), albeit only 5 of the groups have 50 (>1%) or more individuals in them. These five clades are E, G, I, J and R and have 153 (3%), 94 (1.9%), 960 (19%), 142 (2.8%) and 3564 (72%) individuals in them; (**b**) Y-DNA haplogroup frequencies in ALSPAC Many of the individuals had extensive Y-DNA Single Nucleotide Polymorphism (SNP) data (which passed quality control (QC)), which enabled us to pinpoint with more precision which Y-DNA haplogroup they belonged to. Figure 2a shows the most detailed haplogroup determination; and only the ones with over 50 individuals (>1%) are shown. Where the haplogroup branching halts is an indication of how far we could reliably determine the Y-DNA genealogical branch an individual belongs to: R1b1b2h: R-U152; R1b1b2g1: R-U198; R1b1b2g: R-U106; R1b1b2e: R-M222; R1b1b2d: R-SRY_2627_; R1a1: R-M17/M198; J2: J-M172; I1c: I-P109; G2a: G-P15; E1b1b1a2: E-V13.

**Figure 2:**
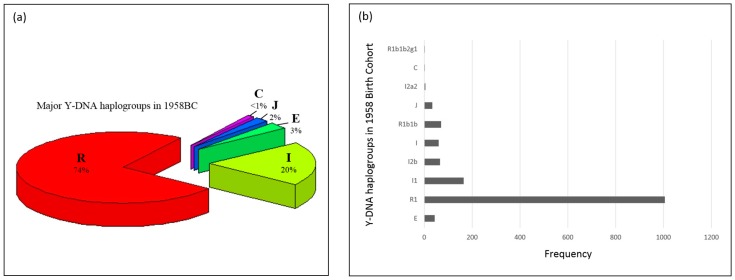
(**a**) Y-DNA haplogroups in 1958 Birth Cohort (Dataset: EGAD00000000022). The 1958 Birth cohort has individuals belonging to five major Y-DNA haplogroups (E, I, J, C, R), albeit two of the groups have less than 50 individuals in them. The clades E, I, J, C and R have 44 (3%), 296 (20%), 34 (2%), 1 (<0.1%) and 1078 (74%) individuals in them; (**b**) Y-DNA haplogroup frequencies in 1958BC (Dataset: EGAD00000000022) Similar to Figure 1a, 1958BC provides dense SNP data which enabled deeper haplogroup determination. The frequencies of haplogroups are 1, 1, 5, 34, 71, 60, 66, 165, 1006 and 44. R1b1b2g1: R-U198; I2a2: I-M26; R1b1b: R-P297.

Figure 1a presents the Y-DNA haplogroups observed in ALSPAC. Y-DNA Clade/Haplogroup R is the most frequent (72%) and I the second most common Y-DNA haplogroup (19%). Y-DNA haplogroups subclades observed in ALSPAC are shown in Figure 1b. Most of the males in ALSPAC belong to the R1b1b2 (R-M269) haplogroup (over 3400 individuals) which is also one of the most common haplogroups in Europe [35]. Figure 2a,b presents the Y-DNA haplogroup profile of 1958BC. Five Y-DNA haplogroup were observed. Similar to ALSPAC, haplogroup R was the most frequent (74%), followed by haplogroup I (20%).

There was not enough evidence for association between Y-DNA haplogroups (using haplogroup R as baseline) and BMI (*p*-value = 0.066) in ALSPAC (Table 1) and in the 1958 cohort (*p*-value = 0.107) (Table 1).

Summary statistics of the BMI observed for the two main Y-DNA haplogroups in ALSPAC and 1958BC can be found in Table 2.

Table 3 includes 32 SNPs previously reported to be associated with BMI [34] and presents the association between each SNP and BMI observed for individuals belonging to Y-DNA haplogroups I and R in ALSPAC.

**Figure 3:**
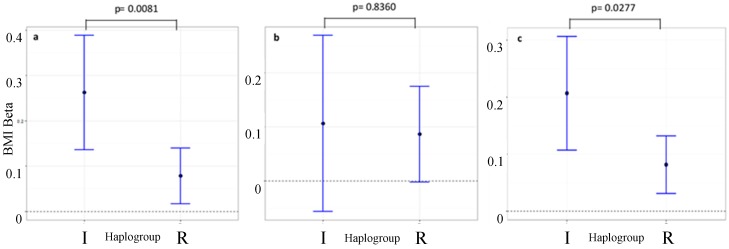
Subgroup analysis comparing effect size of rs8050136 on BMI in two Y-DNA haplogroups. Results from (**a**) ALSPAC (**b**) 1958BC (**c**) ALSPAC and 1958BC combined. The statistics above represent *p*-values from the likelihood ratio test for interaction between Y-DNA haplogroup I and rs8050136. Heterogeneity tests (*z*-test) comparing Y-DNA haplogroups I and R yielded *p*-values of 0.005, 0.4169 and 0.014 for (**a**–**c**) respectively. ggplot2 package in R [36] was used to create the plot.

Table 3 also shows the results from the heterogeneity test (*z*-test) used to compare the effect sizes derived from the two Y-DNA haplogroups. Only one instance of heterogeneity between the two haplogroups was observed after adjusting for Bonferroni correction which was observed for rs8050136 in *FTO* which yielded a *z*-heterogeneity test *p*-value of 0.005. The likelihood ratio test for interaction between the same SNP and haplogroup I yielded a similar *p*-value of 0.008 (Figure 3a). In ALSPAC, there was a difference in the effect size of this SNP within haplogroup I (*p* = 7.00 × 10^−5^, β = 0.266, Standard Error = 0.066, *n* = 508) compared with haplogroup R (*p*-value = 1.4 × 10^−2^, β = 0.079, Standard Error = 0.032, *n* = 1965).

The *p*-value for heterogeneity (as measured by the likelihood ratio test) between Y-DNA haplogroups I and R in relation to the association between rs8050136 (in *FTO*) and BMI was 0.008. To test whether the possible differential effect of the *FTO* SNPs replicate in another cohort, the top-hit rs8050136 SNP was analysed in the 1958BC and the results are presented in Table 4. The likelihood ratio test for interaction (and *z*-heterogeneity test) yielded a *p* > 0.05 (*p*-value = 0.836, *z*-test *p*-value = 0.417, see Figure 3b). For sensitivity, we repeated the same analysis using eight other SNPs in linkage disequilibrium (LD) with rs8050136 and the results were consistent for all nine *FTO* SNPs, with heterogeneity between Y-DNA haplogroups I and R in all cases (Table 5). As a sensitivity analysis, we repeated the interaction analysis for rs8050136, this time including the individuals in the “J” clade with the “I”s. The likelihood ratio test for these analyses resulted in a *p*-value of 0.0069 (‘IJ’ group sample size = 589)—with very similar β (0.253—see Haplogroup I result for rs8050136 in Table 3) and consistent direction of effect.

## 4. Discussion

Population stratification is a potential confounder in genetic association studies. Haplotypic variation and sub-clustering can still be present even after accounting for PCs (see reference [4] for an example). Therefore, an apparently homogeneous population (defined by PCA) can harbour different subgroups of individuals. In this work, we analysed whether this was the case for Y-DNA haplogroups.

We used the ALSPAC cohort—formed of a relatively homogeneous group of participants—for proof of concept that Y-DNA haplogroup variation is present even after accounting for PCs. We then looked to see whether this variation could confound the genetic association studies related to BMI. In this work, we also present the Y-DNA haplogroup profiles of two cohorts for genetic epidemiological studies—ALSPAC and the 1958BC. Within a homogenous looking population there were individuals belonging to different paternal lineages. We undertook a stratified analysis of Y-DNA haplogroups in ALSPAC. This can be the case especially if the trait is associated to the haplogroup(s). In this study we observed no conclusive evidence for differences in SNP/BMI association according to Y-DNA haplogroups in either ALSPAC or the 1958BC.

A key aspect about the relevance of the Y-DNA structure is that there can be an effect on the phenotype associations if the structure is also correlated with BMI and if the actual haplogroup interacts directly with the assessed gene variants. Alternatively, other loci would be enough to obscure inference. Our study showed no clear evidence of this correlation and interaction. However, one could argue that the lack of replication could be explained by heterogeneity in the two datasets (ALSPAC and 1958 cohort), since the former includes children and the latter, adults. Therefore, the differences in betas and interaction effects could be due to other differences between the cohorts.

**Figure 4:**
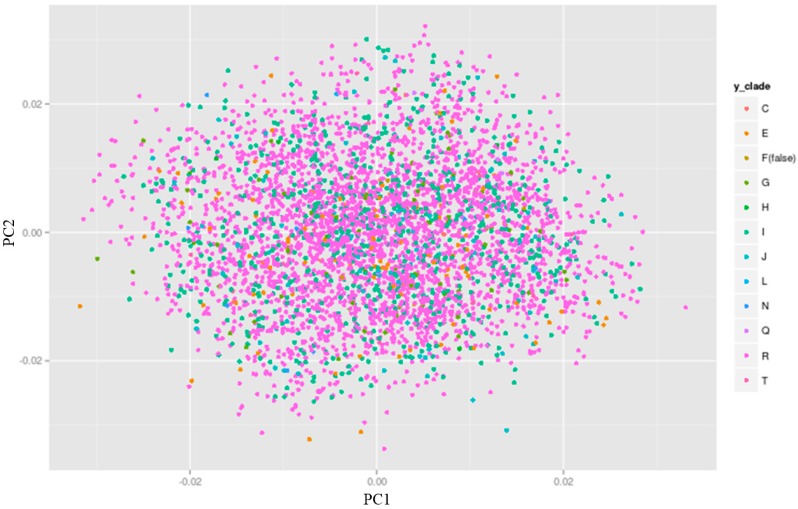
Y-DNA haplogroup vs top two principal components (PCs) in ALSPAC individuals plotting the Y-DNA haplogroup clades on a principal component analysis (PCA) plot reveals that there is no apparent sub-clustering within the ALSPAC individuals. Thus, adding Y-DNA haplogroup information as covariates to control for additional population stratification in ALSPAC is not needed. Ggplot2 package in R [36] was used to create the plot.

A sub-clustering due to Y-DNA haplogroups can be revealed by plotting the Y-DNA haplogroup information versus the top two PCs on a scatter plot (see ALSPAC example on Figure 4). For the ALSPAC cohort, sub-clustering due to Y-DNA haplogroups could not be observed thus adding Y-DNA haplogroups as covariates in a genetic association study is not essential (Figure 4). However, there may be cases and cohorts where the contrary is true, thus an additional check on this can eliminate subtle population stratification due to non-recombining paternal ancestry of individuals within a sample.

A substantial caveat of using Y-DNA haplogroups is the exclusion of females in the analyses. However, this limitation is not present for mitochondrial DNA haplogroups, which requires further study in this regard. Another caveat is sample size, a problem for many European Y-DNA haplogroups, especially in the deeper sub-branches of the Y-DNA genealogical tree. The idea of using Y-DNA haplogroup information to inform genetic association studies is still underexplored and requires further research using different traits and haplogroups.

Overall, although Y-DNA haplogroup sub-structure could be a problem theoretically (and in some populations more than others), our results are in accordance with evidence showing that gross structure in common variant analysis does not seem to be a problem after PCA. On the other hand, recent evidence suggests that finer structure does exist for people of the British Isles [4] and is most probably true for many other populations. It follows that if stratification is not really a problem, further studies could be efficiently improved by capturing some of this finer structure. This could be partially explained by variation of Y-DNA haplogroups (see Supplementary Materials for further discussion).

Our initial results can be explained by chance and hence was not replicated in the replication dataset. However, it also illustrates the potential of Y-DNA haplogroup data which could be tested in phenome-wide association studies (PheWAS) settings to systematically assess the impact of substructure.

## Figures and Tables

**Table 1:** Linear regression between body mass index (BMI) and Y-DNA haplogroup I in two cohorts—Avon Longitudinal Study of Parents and Children (ALSPAC) and 1958BC. The *z*-test for heterogeneity shows that the effect size of Y-DNA haplogroup I on BMI is differential depending on the cohort. Std. Error: standard Error, CI: confidence interval.

Cohort Name	*n*	β	Std. Error	95% CI	*p*-Value	Z-Test for Heterogeneity
ALSPAC	2817	−0.085	0.046	−0.175–0.006	0.066	z = 2.3748 *p*-value = 0.0088
1958BC	1351	0.106	0.0660	−0.023–0.236	0.107	

**Table 2:** Summary statistics of the two Y-DNA haplogroups for BMI in ALSPAC. Std. Dev: Standard Deviation.

Variable	*n*	Mean Age (Range)	Mean BMI	Std. Dev	Min BMI	Max BMI	Std. Error	95% CI for Mean
Y-DNA I—ALSPAC	583	7.57 (7.07–9.49)	16.02	2.018	12.36	28.28	0.084	15.86–16.19
Y-DNA R—ALSPAC	2234		16.12	1.843	11.78	27.28	0.039	16.04–16.20
Y-DNA I—1958BC	293	23.0 (22.9–23.1)	23.32	2.981	18.02	39.20	0.174	22.98–23.66
Y-DNA R—1958BC	1058		23.00	2.790	14.00	37.32	0.086	22.83–23.17

**Table 3:** Comparison of associations observed between ALSPAC individuals with Y-DNA haplogroup I and Y-DNA haplogroup R for Single Nucleotide Polymorphisms (SNPs) previously reported to be associated with BMI. β-Coef: beta coefficient (i.e., effect size).

SNP ID	Nearby Gene	Haplogroup I (508 Individuals)			Haplogroup R (1965 Individuals)			Test of Heterogeneity (*z*-Test *p*-Value)
		*p*-Value	β-coef.	Std. Error	*p*-Value	β-coef.	St. Error	-
rs8050136	*FTO*	5.45 × 10^−5^	0.261	0.064	1.60 × 10^−2^	0.076	0.032	0.0050
rs2815752	*NEGR1*	0.748	0.021	0.064	0.454	0.023	0.031	0.9776
rs1514175	*TNNI3K*	0.436	0.050	0.064	0.425	0.025	0.031	0.7252
rs1555543	*PTBP2*	0.392	0.058	0.068	0.225	0.039	0.032	0.8004
rs543874	*SEC16B*	3.00 × 10^−3^	*−0.230*	0.077	1.10 × 10^−2^	*−0.097*	0.038	0.1214
rs2867125	*TMEM18*	1.50 × 10^−2^	0.207	0.085	1.00 × 10^−3^	0.130	0.040	0.4124
rs713586	*RBJ*	0.410	0.054	0.066	4.60 × 10^−2^	0.061	0.031	0.9235
rs887912	*FANCL*	0.618	*−0.036*	0.071	0.169	0.046	0.033	0.2949
rs2890652	*LRP1B*	0.120	0.138	0.089	0.242	0.047	0.041	0.3531
rs13078807	*CADM2*	0.158	0.108	0.077	0.404	*−0.032*	0.039	0.1048
rs9816226	*ETV5*	0.165	0.123	0.088	6.00 × 10^−2^	*−0.077*	0.041	0.0394
rs10938397	*GNPDA2*	0.551	*−0.039*	0.065	3.00 × 10^−3^	*−0.092*	0.031	0.4617
rs13107325	*SLC39A8*	0.883	0.018	0.122	7.00 × 10^−2^	*−0.106*	0.058	0.3587
rs2112347	*FLJ35779*	0.359	*−0.064*	0.069	0.110	*−0.052*	0.033	0.8753
rs4836133	*ZNF608*	0.497	*−0.047*	0.068	0.925	*−0.003*	0.031	0.556
rs206936	*NUDT3*	0.401	*−0.070*	0.083	0.986	*−0.001*	0.039	0.4518
rs987237	*TFAP2B*	0.523	*−0.056*	0.087	3.80 × 10^−2^	*−0.082*	0.039	0.7851
rs10968576	*LRRN6C*	3.90 × 10^−2^	*−0.143*	0.069	0.782	0.009	0.033	0.0469
rs4929949	*RPL27A*	0.546	0.040	0.066	0.968	0.001	0.031	0.5928
rs10767664	*BDNF*	0.972	0.003	0.078	0.136	0.055	0.037	0.547
rs3817334	*MTCH2*	0.563	*−0.040*	0.069	0.863	0.005	0.031	0.5519
rs7138803	*FAIM2*	0.874	0.010	0.065	9.00 × 10^−3^	0.085	0.033	0.3036
rs4771122	*MTIF3*	0.583	*−0.044*	0.079	4.90 × 10^−2^	*−0.074*	0.038	0.7322
rs11847697	*PRKD1*	0.549	*−0.106*	0.176	1.00 × 10^−3^	*−0.234*	0.072	0.5009
rs10150332	*NRXN3*	0.764	0.023	0.076	0.836	−0.008	0.038	0.7152
rs2241423	*MAP2K5*	8.50 × 10^−2^	*−0.130*	0.075	0.818	0.009	0.038	0.0983
rs12444979	*GPRC5B*	0.967	0.004	0.094	2.00 × 10^−3^	0.133	0.043	0.212
rs7359397	*SH2B1*	5.20 × 10^−2^	*−0.127*	0.065	0.602	*−0.016*	0.031	0.1232
rs571312	*MC4R*	0.199	0.099	0.077	2.00 × 10^−3^	0.116	0.037	0.8423
rs29941	*KCTD15*	0.233	0.087	0.073	0.114	*−0.051*	0.032	0.0834
rs2287019	*QPCTL*	0.479	0.056	0.078	0.801	*−0.010*	0.040	0.4515
rs3810291	*TMEM160*	0.218	0.099	0.080	0.257	0.042	0.037	0.5178

**Table 4:** Comparison of the associations observed between 1958BC individuals with Y haplogroup I and Y haplogroup R for the only SNP showing a *p*-value ~ 0.05 in any of the two cohorts analysed. β-Coef: beta coefficient (i.e., effect size); St. Error: Standard error.

SNP ID	Nearby Gene	Haplogroup I (293 Individuals)			Haplogroup R (1058 Individuals)			Test of Heterogeneity
		*p*-Value	β-coef.	St. Error	*p*-Value	β-coef.	St. Error	
rs8050136	*FTO*	0.201	0.106	0.083	0.056	0.086	0.045	*p*-value = 0.417

**Table 5:** Comparison of associations observed between ALSPAC individuals with Y haplogroup I and Y haplogroup R for *FTO* SNPs observed in ALSPAC. All *z*-test *p*-values are less than 0.01. β-Coef: beta coefficient (effect size); St. Error: Standard error.

SNP ID	Haplogroup I (521 Individuals)			Haplogroup R (2011 Individuals)			Test of Heterogeneity
	*p*-Value	β-coef.	St. Error	*p*-Value	β-coef.	St. Error	
rs8050136	5.45 × 10^−5^	0.261	0.064	1.60 × 10^−2^	0.076	0.032	I^2^ = 84.9%
rs9940128	1.11 × 10^−4^	0.252	0.065	2.30 × 10^−2^	0.072	0.032	I^2^ = 83.8%
rs9939609	1.11 × 10^−4^	0.252	0.065	2.30 × 10^−2^	0.072	0.032	I^2^ = 83.8%
rs9930506	8.49 × 10^−5^	0.266	0.067	2.20 × 10^−2^	0.075	0.033	I^2^ = 84.7%
rs17817449	1.12 × 10^−4^	0.251	0.065	3.10 × 10^−2^	0.068	0.032	I^2^ = 84.3%
rs7193144	1.11 × 10^−4^	0.252	0.065	2.00 × 10^−2^	0.074	0.032	I^2^ = 83.4%

## Supplementary Materials

The following are available online at http://www.mdpi.com/2073-4425/9/1/45/s1.
