# Supplementary material for: Using Y-Chromosomal Haplogroups in Genetic Association Studies and Suggested Implications
Source: Genes (Basel). 2018 Jan 22;9(1):45. doi: 10.3390/genes9010045 (PMC5793196; doi:10.3390/genes9010045)
Supplement: Supplementary file 1 [file genes-09-00045-s001.pdf]

genes

MDPI

Supplementary to "Using Y-Chromosomal Haplogroups in Genetic Association Studies and Suggested Implications"

A. Mesut Erzurumluoglu [1], Denis Baird [2], Tom G. Richardson [2], Nicholas J. Timpson [2] and Santiago Rodriguez [2*]

[1] Genetic Epidemiology Group, Department of Health Sciences, University of Leicester, Leicester, United Kingdom.

[2] MRC Integrative Epidemiology Unit (IEU), Population Health Sciences, Bristol Medical School, University of Bristol, Oakfield House, Oakfield Grove, Bristol BS8 2BN, United Kingdom.

1. Possible uses of Y-DNA haplogroups in identifying interactions between autosomal SNPs and phenotypes

In the presence of robust and replicated associations, the stratified analysis of Y-DNA haplogroups could be used to: (i) inform genetic association studies by identifying which haplogroup(s) account for the association; (ii) assess the strength of known associations and observe whether it still holds in all Y-DNA haplogroups; (iii) observe an effect modification; and, in relation to Point (iii), (iv) determine whether the effect modification observed gives a hint about gene–environment interactions or epistasis (gene–gene interaction) occurring between the autosomal loci being analysed and the Y-DNA loci associated with the haplogroup(s) in which one observes that effect modification. Differences seen in effect size can be solely due to regional effects and ancestry, thus addition of principal components (PCs) to the linear regression models are required [1]. The theory behind Point (iv) is to use Y-DNA haplogroups as a proxy to see how a mutation (or even a combination of mutations) in the non-recombining region of the Y chromosome affects the effect size of another autosomal SNP. This could give insights into whether the two loci are linked through some pathway. Since many Y-DNA mutations will be rare and not feasible for analysis, we propose using Y-DNA haplogroups as a proxy for common Y-DNA mutations and every variation that they may "tag" (e.g., marker for gene–gene interactions, marker for environmental exposure).

2. Interpretation of differential effect of a SNP(s) due to stratification according to Y-DNA haplogroups

Differential effect of a SNP(s) due to stratification according to Y-DNA haplogroups could be interpreted in three ways. An effect could: (1) reflect gene–gene interactions (epistasis); (2) be marker of an environmental factor; (3) provide evidence for gene–environment interactions (i.e., mediating effect); or (4) represent a new approach to test for homogeneity among populations for fine-scale population stratification when added to principal components. These four possible mechanisms are discussed below:

2.1. Gene–Gene Interaction (Epistasis)

Epistasis refers to the effect of one gene being modified by the action of one or more genes. A modification of the effect sizes between autosomal SNPs and a phenotype could be mediated by the type of Y-DNA haplogroup. A possible mechanism to account for this is gene–gene interaction between the SNP(s) analysed and the variants associated with the Y-DNA haplogroup, which brings about the change in effect size. In this way, the association between an autosomal SNP(s) and a phenotype could be mediated by genetic variation on the Y chromosome, that is, by the type of Y-DNA haplogroup the individuals belong to. How genetic variation on the Y chromosome affects the effect size of another autosomal SNP on a phenotype can provide insights into whether the two loci

are linked through some pathway or not. Discovering interaction could also represent unequal distribution of rare alleles that have an effect on the phenotype analysed. To support this hypothesis, formal tests of genetic interaction and ideally further molecular and functional studies (both eluding the scope of this paper) would be required.

2.2. Environmental Factors

Y-DNA haplogroups could be markers of an environmental effect. It is known that different haplogroups have a different origin and history. It is possible that, in the present days, different haplogroups mark epigenetic differences and even environmental differences beyond the effect of genes. Again, this hypothesis will need to be tested with appropriate study designs to be confirmed or refuted.

2.3. Environment–Genetic Interaction (Epigenetic)

A possible underlying mechanism for differences due to Y-DNA haplogroups is the presence of geographical effects (e.g., due to environment–genetic interaction). As mentioned above, the Y-DNA haplogroup can turn out be a marker for environmental effects. However, gene–gene interactions between the Y-DNA SNPs and autosomal SNPs are also a possibility, and identifying the true biology behind such a finding is going to require functional analyses.

2.4. Population Stratification

Tests for population stratification include genomic control [2], principal component analysis [1] and linear mixed models [3]. The analysis of Y-DNA haplotypes in the context of population stratification has already been suggested [4, 5]. However, our approach represents a new dimension to study the effect of population stratification in genetic association studies. Haplogroup information can be added to the principal component used in regression models where sub-clustering is observed. A sub-clustering due to Y-DNA haplogroups can be revealed by plotting the Y-DNA haplogroup information versus the top two principal components on a scatter plot (see ALSPAC example in Figure S1). For the ALSPAC cohort, sub-clustering due to Y-DNA haplogroups could not be observed, thus adding Y-DNA haplogroups as covariates in a genetic association study is not essential (Figure S1). However, there may be cases and cohorts where the contrary is true, thus an additional check on this can eliminate subtle population stratification due to non-recombining paternal ancestry of individuals within a sample.

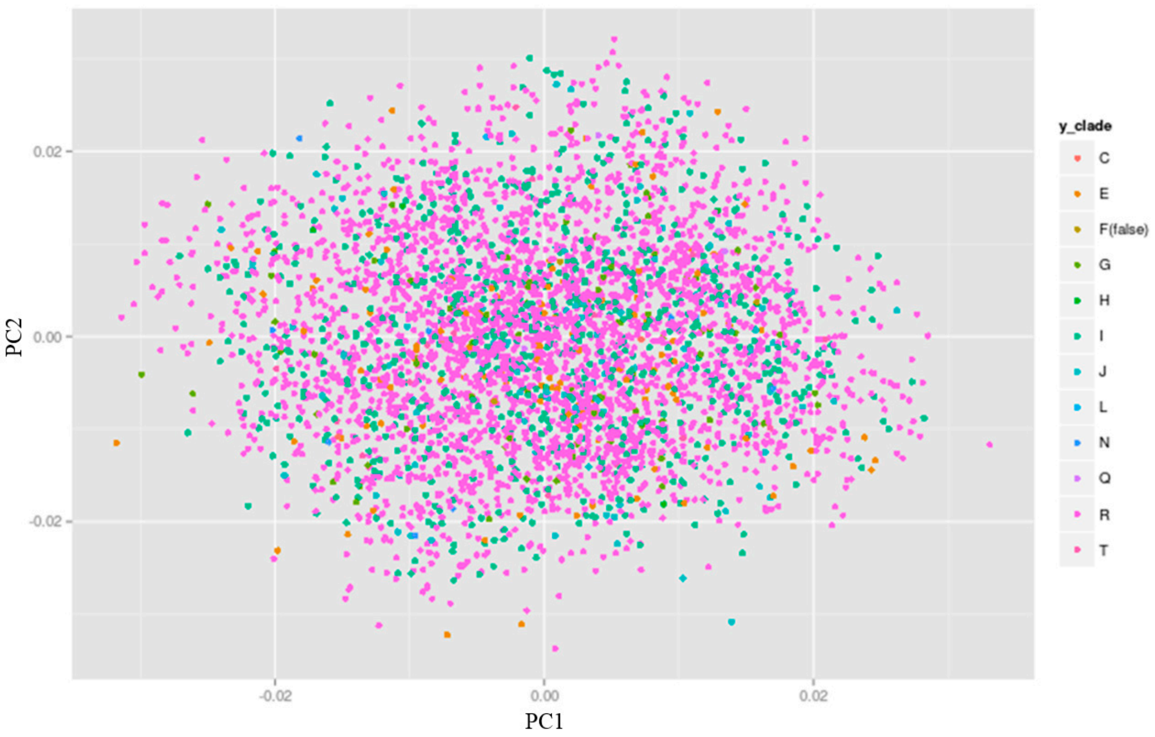

Figure S1. Y-DNA haplogroup vs. top two principal components in ALSPAC individuals. Plotting the Y-DNA haplogroup clades on a PCA plot reveals that there is no apparent sub-clustering within the ALSPAC individuals. Thus, adding Y-DNA haplogroup information as covariates to control for additional population stratification in ALSPAC is not needed. ggplot2 package in R was used to create the plot.
